# Correlation evaluation between cancer microenvironment related genes and prognosis based on intelligent medical internet of things

**DOI:** 10.3389/fgene.2023.1132242

**Published:** 2023-02-09

**Authors:** Shoulei Ren, Wenli Cao, Jianzeng Ma, Hongchun Li, Yutao Xia, Jianwen Zhao

**Affiliations:** ^1^ Oncology Department, Yangguangronghe Hospital, Weifang, Shandong, China; ^2^ Nerosurgery Department, Yangguangronghe Hospital, Weifang, Shandong, China

**Keywords:** tumor microenvironment, internet of things technology, smart healthcare, biological, correlation evaluation

## Abstract

The study of tumor microenvironment plays an important role in the treatment of cancer patients. In this paper, intelligent medical Internet of Things technology was used to analyze cancer tumor microenvironment-related genes. Through experiments designed and analyzed cancer-related genes, this study concluded that in cervical cancer, patients with high expression of P16 gene had a shorter life cycle and a survival rate of 35%. In addition, through investigation and interview, it was found that patients with positive expression of P16 and Twist genes had a higher recurrence rate than patients with negative expression of both genes; high expression of FDFT1, AKR1C1, and ALOX12 in colon cancer is associated with short survival; high expressions of HMGCR and CARS1 is associated with longer survival; overexpression of NDUFA12, FD6, VEZT, GDF3, PDE5A, GALNTL6, OPMR1, and AOAH in thyroid cancer is associated with shortened survival; high expressions of NR2C1, FN1, IPCEF1, and ELMO1 is associated with prolonged survival. Among the genes associated with the prognosis of liver cancer, the genes associated with shorter survival period are AGO2, DCPS, IFIT5, LARP1, NCBP2, NUDT10, and NUDT16; the genes associated with longevity are EIF4E3, EIF4G3, METTL1, NCBP1, NSUN2, NUDT11, NUDT4, and WDR4. Depending on the prognostic role of genes in different cancers, they can influence patients to achieve the effect of reducing patients’ symptoms. In the process of disease analysis of cancer patients, this paper uses bioinformation technology and Internet of things technology to promote the development of medical intelligence.

## 1 Introduction

The correlation between cancer TME related genes and prognosis is an unclear field at present, and there are big problems at present. The data of cancer analysis not only comes from patients’ information, but also needs to be analyzed in combination with patients’ conditions to understand the relationship between genes behind them, so as to achieve a good cure effect and bring convenience to public life. It is necessary to analyze the correlation between cancer TME related genes and prognosis in this field.

TME is a topic of wide scholarly interest and has been studied by a number of scholars in this area. Hu et al. (2020) included 85 patients to investigate the effect of TME and related genes on patients with osteosarcoma. Survival analysis showed that patients with higher immune scores had good overall survival and disease-free survival. In addition, 769 genes were identified as TME-associated genes. It was concluded that TME was associated with the prognosis of patients with osteosarcoma. A prognostic model based on TME-related genes can effectively predict overall survival and disease-free survival in patients with osteosarcoma. Yuan et al. (2020) performed a systematic investigation of TME and identified TME-related genes with prognostic value in patients with lung adenocarcinoma, identifying a total of 281 prognostic TME-related genes. Subsequently, functional analysis and protein-protein interaction network analysis showed that these genes were mainly associated with immune response, inflammatory response and chemotaxis. Luo et al. (2021) performed survival analysis to identify colon cancer pivotal genes and used these pivotal genes with TNM staging to construct prognostic models. In addition, the correlation between pivotal gene expression and immune cell infiltration was assessed. Wang et al. (2021) investigated the potential relationship between co-expression modules and TME by weighted gene co-expression network analysis. Firstly, several TME-related genes were integrated and a risk prediction model was established. The model can accurately predict the progression and prognosis of BLCA and provide clinical implications for risk stratification, immunotherapy drug screening and treatment decisions. Liu et al. (2021) explored the relationship between TME and prognosis of colorectal cancer and identified prognostic genes associated with the microenvironment of colorectal cancer. Gene expression data were collected from the Cancer Genome Atlas and stromal/immune cell scores for colorectal cancer were calculated and their relationship with clinical outcomes. Li et al. (2021a) assessed the relationship between TME and prognosis and explored prognostic genes in rectal cancer. An expression data algorithm was used to estimate stromal and immune cells in malignant tumors to calculate the immune/stromal score. The correlation between immune/stromal score and survival time as well as clinical characteristics was assessed. Zhang et al. (2020) used the expression data algorithm to estimate cells and immune cells in tumor tissue to calculate the immune/stromal score. Patients with clear cell renal cell carcinoma in the Cancer Genome Atlas database were classified into low and high groups based on the score, and genes that were differentially expressed and significantly correlated with prognosis were identified. The results of functional enrichment analysis and protein-protein interaction networks indicated that these genes are mainly involved in the regulation of extracellular matrix and cellular activity. Finally, a series of microenvironment-related genes were obtained, which predict the prognosis of patients with clear cell renal cell carcinoma. In many studies, the analysis steps were similar, all of them investigated the prognosis of cancer, indicating the practical value of the investigation of cancer genes and prognosis.

The IoT technology has many applications in the medical field. Zhu et al. (2019) believed that the development of the IoT advanced medical care, and discussed different aspects of intelligent medical care, as well as health data and patient centered health management. Islam and Rahaman (2020) developed the intelligent medical monitoring system in the IoT environment. Javaid and Khan (2021) analyzed that the medical care enabled by the IoT could help meet the challenge of the COVID-19 pandemic. Kondka et al. (2022) used an intensive healthcare monitoring paradigm based on the IoT machine learning strategy. Zhang et al. (2018) proposed an intelligent IoT connection architecture for smart hospitals based on narrowband IoT, and introduced edge computing to deal with the delay requirements in the medical process. He developed an infusion monitoring system to monitor the real-time decline rate and residual drug volume during intravenous infusion, and discussed the challenges and future direction of building a smart hospital by connecting intelligent things. Li et al. (2021b) made a comprehensive investigation on the intelligent medical system of the IoT by using big data analysis of machine learning. Mohammed (2020) studied the detection and diagnosis system of novel coronavirus using the smart helmet based on the IoT. The analysis of IoT technology has not yet analyzed the correlation between cancer microenvironment related genes and prognosis.

In order to make the concept of intelligent medical care deeply rooted in people’s hearts, this paper analyzed the IoT for intelligent medical care, tumor microenvironment and their applications. The correlation between cancer TME related genes and prognosis based on the intelligent medical IoT was evaluated, and four common cancers were took as examples to analyze their genes and prognosis effects. Finally, the feasibility conclusion was drawn. This paper provided a reference path for the correlation analysis between cancer microenvironment related genes and prognosis.

## 2 Intelligent medical IoT

### 2.1 IoT and intelligent healthcare

The structure of the IoT is very complex and consists of three main parts: The sensor layer is responsible for collecting information (it uses smart cards, Radio Frequency Identification (RFID) tags, two digit barcodes, sensors, etc.); the network layer is responsible for transmitting information (it uses wireless network, cellular network, wired network, RFID network, etc.); the application layer supports information analysis and processing and management decisions (Qadri et al., 2020).

Smart medical care comes into being under specific circumstances: In the new round of medical reform, the old medical system cannot meet the needs of today’s constantly developing society, while the main reason for the new medical reform is that it is difficult to see a doctor and the cost is high. With the change of life style, acute and chronic diseases, aging population, and many hostile environmental threats, the demand for hospital information technology is growing. Intelligent medical management is to optimize the combination of QR code, RFID, wireless network and other technologies with the traditional hospital system integration.

### 2.2 Application of IoT in medical treatment

**FIGURE 1:**
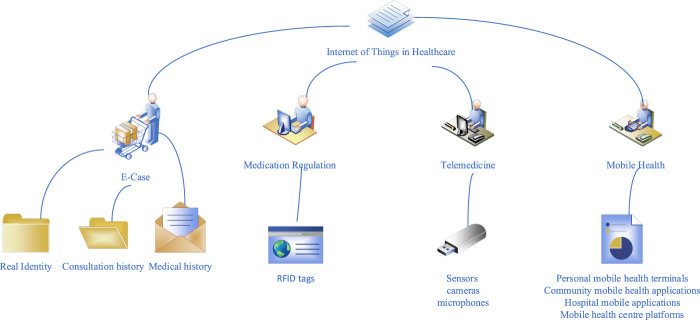
Internet of Things in healthcare.

The IoT is a large-scale network established by connecting various information sensors to the Internet, which collects any object or process that needs real-time tracking, connection and interaction. It also collects sound, light, heat, electricity, mechanical, chemical and bioenergy, location and other important information. The goal is to connect objects to objects, objects to people, and all objects to the network so that they can be more easily identified, managed, and controlled. The IoT is the extension and expansion of computer networks, which is a huge network formed by connecting various information sensors to the Internet. It enables all people and things to connect to the network and manage important events in a timely manner. The IoT has a wide range of applications, including intelligent security, intelligent logistics, intelligent medical and other fields (Habibzadeh et al., 2019). The main applications of IoT technology in the medical field are electronic documents, drug monitoring, telemedicine, etc., as shown in Figure 1.

#### 2.2.1 Electronic case

The electronic case mainly includes checking the patient’s real identity, consulting records, medical history, etc., In emergency situations, doctors can use electronic medical records to ensure efficient and timely care. Electronic records include information collection, storage, processing, and intelligent services. Personal electronic medical records collect and store clinical information of patients in outpatient and ward of different hospitals, as well as electrocardiogram, ultrasound and other examination information. In the consultation process, the patient only needs to bring a card, and the doctor can understand the patient’s full medical history and quickly solve the problem.

#### 2.2.2 Drug supervision

The IoT is increasingly being used in the field of drug monitoring, especially for obtaining information about drug production and circulation, and preventing counterfeiting and abuse (Singh et al., 2020). In recent years, medical malpractice has gradually become a serious problem in society. The workload of medical personnel is increasing, because they must constantly check the patients’ drugs to avoid the wrong patient and drug combination. Traditional barcode recognition has some disadvantages, such as poor readability and reduced readability. Only RFID tags can be used after drug production, which can record all production information and drug flow information. They have tamper proof function. The advantage of RFID tag is that it can store more data and capture obscure objects without touching, so as to make them invalid. They can be used to help staff check drugs and issue alerts to prevent dispensing errors or drug abuse by patients.

#### 2.2.3 Telemedicine

Telemedicine mainly involves remote monitoring and home care. Among them, wireless sensor network technology is used to monitor the physiological state and movement of the monitored object, so as to assist patients in the event of an accident. A telemedicine system usually consists of a ward, a home ward and a dedicated remote ward that can be connected *via* the Internet. In case of an accident, intelligent terminals such as mobile phones and computers can be used to communicate with remote experts in a timely manner. Remote experts can use various physiological information sensors, cameras, microphones, and other equipment to directly monitor and understand the patient’s condition and provide timely treatment. This can save time and prevent patients from going to the hospital blindly.

#### 2.2.4 Mobile medicine

Mobile medical needs a wireless network as the basis of a complete and efficient mobile medical system, including personal mobile medical terminals, community mobile medical applications, hospital mobile applications and mobile medical center platforms (Tian et al., 2019). In the era of limited medical resources, efficient medical resources can be integrated and maximized to better meet the challenges. Nowadays, with the rapid development of communication technology and smart phones, China’s communication network has been greatly expanded. Medical software on smart phones is also being used more and more, and mobile medicine is making remarkable achievements. For example, the deployment, inventory and management of medical equipment or the use of mobile ward rounds, mobile medicine, electronic health records and drug management are becoming more and more popular, and these have also been applied for the first time in rural and remote mountain areas.

The intelligent sign in terminal is very useful for feeding patients, hanging water for nurses and registering doctors. This technology can be used to record patient information, prescription drugs and various physiological parameters. RFID technology can quickly retrieve relevant information through scanning codes and establish personal care standards. It effectively reduces the workload of medical staff and improves the implementation level of health education. Nurses are very important to the information and knowledge in the work process. Now, various types of healthcare management software are widely used in mobile personal healthcare. Although it has not been widely used in China, many problems remain unsolved. However, it is believed that its huge advantages would promote the progress and development of medical and healthcare.

## 3 Tumor microenvironment and its application

### 3.1 Tumor microenvironment

TME refers to the internal environment where tumor cells originate and live. This includes not only the tumor cells themselves, but also their environment, fibroblasts, immune and inflammatory cells, glial cells, and other cells, as well as the stroma, microvessels, and biomolecules that infiltrate near the tumor. The most obvious signs of TME are hypoxia, low pH and high pressure. With the help of TME, tumors weaken anti-tumor immune response, and maintain proliferation, which also prevent apoptosis and maintain inflammatory environment and angiogenesis. The change of immune surveillance function from tumor clearance to tumor induction is a complex process, which involves several signal pathways mediated by cytokines expressed by tumor cells, immune, cells and other non-tumor cells, such as tumor related epithelial cells or fibroblasts in surrounding tissues.

The basis of tumor immune monitoring is fibroblasts, tumor related macrophages, tumor related neutrophils, bone marrow derived suppressor cells, tumor related fibroblasts, regulatory T cells and other cells that change the balance of immune cells in TME. This leads to increased inflammation and angiogenesis; phenotypic changes of neutrophils from N1 to N2, macrophages from MI to M2, and T cells from Thl to Th2; the number and activity of cytotoxic T cells and antigen presenting cells decreased. The sharp decrease of mature dendritic cells leads to the increase of monocyte progenitor cells, which helps to increase tumor related macrophages and bone marrow derived suppressor cells. The cytokine network formed between these immune cells is mutually reinforcing, which helps maintain the number of TME immune cells that contribute to tumor formation. In addition, transforming growth factor β, vascular endothelial growth factor, chemokines and inflammatory cytokines (especially Th2 mediated cytokines) have been proved to be involved in angiogenesis, inflammation, and tumor immunosuppression. Fibroblasts and regulatory B cells seem to be involved.

### 3.2 Proto oncogene and tumor suppressor gene

#### 3.2.1 Cancer gene

Cancer genes, also known as proto oncogenes, mainly refer to DNA nucleotide sequences that have the ability to cause malignant transformation of cells, that is, the nucleic acid part that causes malignant transformation of cells. This concept was first discovered in the study of retroviral RNA viruses, which were initially called viral tumor genes. Later, it was found that the reverse transcriptional RNA of this nucleotide sequence also existed in the cell gene, and even found the carcinogenic base sequence that did not exist in the virus, so it was renamed as the cell tumor gene. Tumor gene is a highly conserved gene, which is expressed only during embryonic development. In adulthood, it would no longer express itself and would be silent. Tumor genes play an important role in regulating normal cell growth, proliferation, development, and differentiation during embryonic development. Therefore, oncogenes in cells are not only the cause of cancer, but also the genes necessary for life. Oncogenes can be produced only when the mutation of oncogene causes changes in its normal structure and function.

Cancer genes are usually different from normal genes in the following aspects: The expression level of tumor genes is often higher than that of proto oncogenes (non-mutated normal genes corresponding to tumor genes), and sometimes unnatural transcription occurs in differentiated cells. This difference in structure and function often leads to the loss of fusion protein activity, which leads to the imbalance of oncogene proteins and promotes unlimited cell proliferation and abnormal transformation. The multi site mutation of many carcinogenic genes results in the replacement of a single amino acid of the corresponding protein by other amino acids.

#### 3.2.2 Tumor suppressor gene

Tumor suppressor genes are genes that inhibit excessive cell division, thus preventing the development and deterioration of cancer. This is because the normal function of its expression products would be to negatively stimulate cell growth and inhibit proto oncogenes. When carcinogenic genes are destroyed by internal or external factors during cell division and proliferation, such destruction would lead to partial or even complete deletion of genes, thus leading to loss of gene expression or inactivation of gene expression products. As a result, the product of gene expression lost its function, leading to the occurrence of cancer. Tumor suppressor genes generally have the following characteristics: The inhibition of tumor suppressor genes is often accompanied by non-functional mutations involving loss of heterozygosity; tumor suppressor genes often mutate in cancer prone syndromes; somatic mutation occurs in spontaneous tumor suppression; tumor suppressor genes can also inhibit cell growth *in vitro*. Tumor suppressor genes have many important functions. For example, they induce cell differentiation and maintain genetic stability, which also induce cell aging and normal programmed cell death and regulate cell growth. They also inhibit protease activity and change methylase activity, which also regulate histocompatibility antigens and blood vessels and promote the formation of cells. The inactivation of these functions or the deletion or mutation of these genes can lead to the transformation of malignant cells, thus leading to the occurrence of cancer.

### 3.3 Cytokines

**FIGURE 2:**
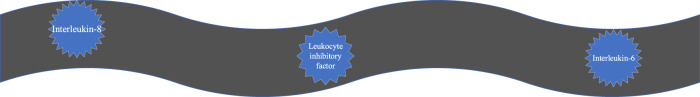
Cytokines.

Many cytokines form a complex network to regulate the immune response of the human body in the local tumor microenvironment, and promote or inhibit the growth, invasion or metastasis of tumors. Cytokines mainly include interleukin-8, leukocyte inhibitory factor and interleukin-6, which are summarized in Figure 2.

#### 3.3.1 Interleukin-8

Interleukin-8 belongs to the CXC chemokine group, which is involved in neutrophil activation and blood taxis. Interleukin-8 plays an autocrine role in pancreatic cancer tissue and stimulates vascular growth, which provides nutrition for pancreatic cancer cells and promotes the proliferation and invasion of pancreatic cancer cells.

#### 3.3.2 Leukocyte inhibitory factor (LIF)

LIF is a growth factor that causes pancreatic cancer canceration. It promotes the proliferation of human pancreatic cancer cells by expressing c-fos gene, Jun-B gene and cyclin E. Clinically, the content of LIF in quasi pancreatic cancer cell line Hs-700T is low, which can induce STAT3 phosphorylation. However, LIF mediated pathway can be inhibited by protein kinase C, protein tyrosine kinase and calmodulin inhibitor.

#### 3.3.3 Interleukin-6 (IL-6)

IL-6 is highly expressed in the matrix of many malignant tumors, so it is an important factor in the relationship between inflammation and tumor. In addition, IL-6 protects tumor cells from DNA damage, oxidative stress and apoptosis caused by treatment by promoting repair and inducing anti apoptosis pathway, which has anti-cancer effect. Therefore, the method of blocking IL-6 alone or in combination with traditional cancer therapy may be a potential therapeutic strategy for the cancer where IL-6 signaling is dominant. IL-6 is highly expressed in some cancers, including breast cancer, lung cancer, stomach cancer, liver cancer and ovarian cancer. The high expression of IL-6 is associated with poor prognosis and can be used as a diagnostic marker of inflammation and malignant tumor.

## 4 Cancer microenvironment related genes based on intelligent medical IoT

The data in this paper were from the database of tumor genome map. The physical condition and physical health of cancer patients received extensive attention. In order to analyze cancer TME related genes, this paper used intelligent medical IoT technology to analyze the patient’s physical data, and selected several common cancers for correlation analysis between cancer TME related factors and prognosis. The cancers investigated in this paper are summarized in Table 1.

**TABLE 1:** Cancers investigated in the paper.

Cancer type	Concept
cervical cancer	Early stage cervical cancer often has no obvious symptoms and signs, and the cervix may be smooth or difficult to distinguish from cervical columnar epithelial ectopic
Colon cancer	A common malignant tumour of the gastrointestinal tract occurring in the colon
Thyroid adenocarcinoma	Malignant tumours arising from the follicular or parafollicular epithelium of the thyroid
Liver Cancer	Malignant tumours of the liver

### 4.1 Cervical cancer

The gene expression profile and clinical follow-up data of 20 cervical cancer patients were downloaded from the tumor genome map database, and the messenger RNA (mRNA) data of 188 cervical cancer patients with complete clinical follow-up information were downloaded from the two microarray datasets of the gene expression comprehensive database.

TISIDB database is an integrated storage portal for interaction between tumor and immune system. This study first determined the common prognostic related DEGs of TCGA dataset and GEO dataset, and then used TISIDB database to evaluate the interaction of common prognostic related DEGs in tumor and immune system.

**FIGURE 3:**
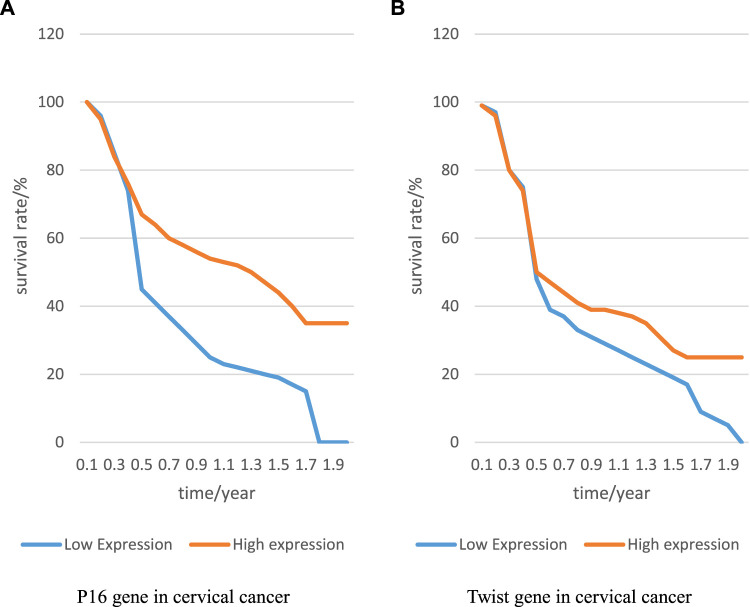
p6 and Twist genes and prognosis of cervical cancer. **(A)** P16 gene in cervical cancer, and **(B)** Twist gene in cervical cancer.

The expression of P16 and Twist genes in cervical cancer was closely related to prognosis. This paper analyzed this cancer to explore the relationship between the expression of P16 and Twist genes and prognosis, and recorded the results to Figure 3:

In Figure 3A represents the relationship between P16 gene expression and patient survival, and B represents the relationship between Twist gene and patient survival. Compared with low expression of P16 gene, the life cycle of patients with high expression of P16 gene was prolonged, and it remained at 35% level; compared with low expression of Twist gene, the life cycle of patients with high expression of Twist gene was also prolonged, indicating that both genes had a great relationship with the prognosis of patients. Through the investigation and interview of patients, it was found that the recurrence rate of patients with positive expression of both genes was higher than that of patients with negative expression of both genes. This showed that if P16 gene was not controlled, cervical cancer would recur. Therefore, it is a target for the prognosis of cervical cancer. The presence or absence of Twist gene expression is related to the prognosis of patients and can be used as a preliminary prognostic marker.

### 4.2 Colon cancer

In this paper, the gene expression profile and clinical follow-up data of 20 colon cancer patients were downloaded from the tumor genome map database, and the survival results of five genes with similar prognostic value were recorded in Table 2:

**TABLE 2:** Prognostic value of colon cancer genes.

Gene type	High expression is associated with shorter survival	High expression associated with prolonged survival
FDFT1	Yes	No
HMGCR	No	Yes
CARS1	No	Yes
AKR1C1	Yes	No
ALOX12	Yes	No

Among the five genes related to the prognosis of colon cancer investigated, the high expression of FDFT1, AKR1C1, ALOX12 was related to the short survival period; high expression of HMGCR and CARS1 was associated with longer survival. The expression and function of genes had a very important relationship with the growth period of patients. Only by mastering the characteristics of different genes could the patient’s symptom response be analyzed, thus bringing advantages to reduce the patient’s symptoms. It could promote the low expression of FDFT1 gene, AKR1C1 gene, and ALOX12 gene, so as to improve the survival of patients. This kept HMGCR gene and CARS1 gene highly expressed to improve the survival of patients.

### 4.3 Thyroid carcinoma

In this paper, the gene expression profile and clinical follow-up data of 20 patients with thyroid cancer were downloaded from the tumor genome map database, and the survival results of 12 genes with similar prognostic value were recorded in Table 3.

**TABLE 3:** Prognostic value of thyroid cancer genes.

Gene type	High expression is associated with shorter survival	High expression associated with prolonged survival
NDUFA12	Yes	No
NR2C1	No	Yes
FD6	Yes	No
VEZT	Yes	No
GDF3	Yes	No
FN1	No	Yes
PDE5A	Yes	No
GALNTL6	Yes	No
OPMR1	Yes	No
IPCEF1	No	Yes
AOAH	Yes	No
ELMO1	No	Yes

Among the 12 genes related to the prognosis of thyroid cancer investigated, the overexpression of NDUFA12, FD6, VEZT, GDF3, PDE5A, GALNTL6, OPMR1, AOAH was related to the shorter survival period; the high expression of NR2C1, FN1, IPCEF1, and ELMO1 was related to the longer survival period.

### 4.4 Liver cancer

In this paper, the gene expression profile and clinical follow-up data of 20 patients with liver cancer were downloaded from the tumor genome map database, and the survival results of 15 genes with similar prognostic value were recorded in Table 4.

**TABLE 4:** Prognostic value of liver cancer genes.

Gene type	High expression is associated with shorter survival	High expression associated with prolonged survival
AGO2	Yes	No
DCPS	Yes	No
EIF4E3	No	Yes
EIF4G3	No	Yes
IFIT5	Yes	No
LARP1	Yes	No
METTL1	No	Yes
NCBP1	No	Yes
NCBP2	Yes	No
NSUN2	No	Yes
NUDT10	Yes	No
NUDT11	No	Yes
NUDT16	Yes	No
NUDT4	No	Yes
WDR4	No	Yes

Among the genes related to the prognosis of liver cancer, the genes related to the shorter survival period were AGO2, DCPS, IFIT5, LARP1, NCBP2, NUDT10, and NUDT16. The genes related to long survival period included EIF4E3, EIF4G3, METTL1, NCBP1, NSUN2, NUDT11, NUDT4, and WDR4.

## 5 Conclusion

In order to analyze the correlation between cancer TME related genes and prognosis, this paper first introduced the intelligent medical IoT, and analyzed the application of the IoT in medicine. Subsequently, TME and its application were introduced. Later, the experiment was designed to analyze the prognosis related genes of patients with cervical cancer, colon cancer, thyroid cancer, and liver cancer. The conclusion was drawn. The relationship between different TME genes and prognosis was very different. The analysis of TME can improve the effect of reflecting cancer analysis, so when analyzing genes, it is necessary to grasp the characteristics of gene expression for cancer diagnosis. This analysis method can improve the prognosis and has potential value for cancer diagnosis and treatment. The use of IoT technology in biomedicine could greatly enhance the efficiency and quality of disease research, thus improving the analysis rate of cancer. In the future, the concept of intelligent medical treatment would gradually become popular. The use of novel research technologies and convenient research methods in this field could greatly improve the effect of cancer analysis, so as to promote the public to keep healthy and meet their medical needs.

## Data Availability

The original contributions presented in the study are included in the article/supplementary material, further inquiries can be directed to the corresponding author.
